# Music: Supporting the Sense of Self in People Living with Alzheimer’s Disease

**DOI:** 10.1192/bjo.2026.11077

**Published:** 2026-06-30

**Authors:** Shriya Karlapudi, Jonathan Corcoran

**Affiliations:** 1 King’s College London–Faculty of Life Sciences and Medicine, London, United Kingdom; 2 Wolfson Sensory, Pain and Regeneration Centre, King’s College London, London, United Kingdom

## Abstract

**Aims:**

Alzheimer’s disease (AD) is a progressive neurodegenerative disorder characterised by cognitive decline, disruption of autobiographical memory and behavioural and psychological symptoms of dementia (BPSD), including anxiety, agitation, apathy and social withdrawal. This significantly affects quality of life and ethical care delivery in clinical and residential settings. Pharmacological treatments provide modest, time-limited benefits and are associated with adverse effects. Consequently, there is growing interest in non-pharmacological, person-centred interventions like music. However, its impact on behaviour, well-being and identity is relatively unexplored.

This paper evaluates music as a person-centred intervention for improving emotional well-being, reducing agitation and supporting a sense of self-identity in people living with AD. It hypothesises that music actively facilitates autobiographical memory recall rather than merely reflecting passive preservation of musical pathways by AD pathology.

**Methods:**

This narrative review synthesised neurobiological, neuroimaging and behavioural studies examining the relationship between music-evoked autobiographical memory, emotional processing and self-identity in AD. Particular attention was paid to disease severity, distinctions between episodic and semantic memory and implications for care homes, specialist services and community-based practice.

**Results:**

Neuroimaging studies have shown involvement of the anterior cingulate cortex, medial prefrontal cortex and hippocampus in both music processing and autobiographical memory retrieval, supporting the existence of specific neural networks for self-identity activated by music. Evidence of music-related hippocampal activation, despite early atrophy in AD, suggests that music may actively preserve autobiographical memory pathways.

Music preferentially enhances episodic and self-defining memory recall, likely through emotional arousal, anxiety reduction and familiarity. Personalised interventions, including self-selected playlists or songs associated with positive emotions, resulted in stronger recall than researcher-selected music. In contrast, semantic memories were less influenced by music, which could create a dissociation between autobiographical memory and self-identity in advanced AD, limiting its effectiveness in later stages. Nevertheless, music reliably improves mood, reduces agitation and enhances social engagement across disease stages.

**Conclusion:**

Music’s effects on autobiographical memory in AD are likely multifactorial and severity-dependent. Although music may not restore self-identity in advanced AD, it improves emotional well-being, reduces agitation and enhances quality of life. Given its non-invasive nature, absence of adverse effects and clinical benefits, music represents an ethically justified and implementable intervention for individuals living with AD. Furthermore, thisresearch suggests the possibility of other sensory cues like sounds, smells, tastes and environments being able to trigger autobiographical memories, highlighting a promising avenue for future research into non-pharmacological, person-centred interventions in AD.

